# An Essay on Curvatures and Diseases of the Spine, Including All the Remote or Spinal Distortions: To Which the Fothergillian Gold Medal Was Awarded by the Medical Society of London

**Published:** 1845-03

**Authors:** 


*An Essay on Curvatures and Diseases of the Spine, including all the remote or spinal distortions: to which the Fothergillian Gold Medal was awarded by the Medical Society of London.* By R. W. Bampfield, Esq., one of the Surgeons to the Royal Metropolitan Infirmary for Diseases of Children, etc. etc. *Edited by* J. K. Mitchell, M. D., Professor of the Practice of Medicine in the Jefferson Medical College of Philadelphia, etc. etc. 8vo., pp. 223. Philadelphia: Ed. Barrington & Geo. D. Haswell, 1845.

Although the Essay which constitutes the basis of the present work, was presented to the Medical Society of London so long ago as 1823, the author has added materially to its value by gleanings from the more recent publications of Dods, Shaw, Jarrold, Charles Bell, Ollivier, and others, so that it may be regarded as the best exposition of the views entertained by the profession at the present day, on the interesting topics of which it treats. The present edition has the advantage, furthermore, of the supervision and occasional notes of a distinguished practitioner, who has had large experience in this class of diseases. Beside an Appendix of Cases, the work is divided into *fourteen Chapters*, which treat of: *Diseases of the Spine; Excurvation of the Spine; Remote and immediate Causes of Curvatures of the Spine, and predisposition to them; Prognosis and Diagnosis; Treatment of Excurvation; Incurvation of the Spine; of Lateral Curvature; Treatment of Lateral Curvature; Angular projection of the Spine; Fractures and Dislocations of the Vertrebræ; Concussions and Stretchings of the Spinal Marrow; Spina-Bifida, or Hydrorachitis; Inflammation of the Medulla Spinalis and its Membranes; some other affections* of the Spine, and diseases said to *originate in its derangement.*" Thus it will be seen that Mr. Bampfield has embraced in his work most of the points of interest connected with Diseases of the Spine, and it is satisfactory to find that the views he has inculcated are in accordance with the observations and experience of the best writers on the same subjects. We are especially pleased to observe that he attaches proper importance to exercise in the open air, and the general hygienic means of preventing deformities, and the other deplorable evils that spring from spinal derangements. In treating of lateral curvature, the author makes the following judicious remarks:

"Some fashionable follies of modern education are justly accused of producing this species of curvature, especially in young females, first, by establishing habits that deprive the muscles of the back of their natural exercise and actions, and thus prevent the attainment of their natural strength, or, by their inaction, induce debility; and, secondly, whilst in this state of debility, by endeavouring to subject the spinal muscles to more exertion than they are capable of using, in the injunctions they receive, to keep the body constantly erect, in the standing and sitting attitudes. Thus the spinal muscles of young females are doomed to inaction, by the trunks of their bodies being imprisoned in stiff stays, or their movements abridged and confined by the use of collars, braces, backboards, or by being stretched motionless on reclining boards, or school-room floors;—or they are subjected to long continued exertion, and the use of one posture, which all our muscles abhor, and soon become weary of, by being placed in education chairs or stools, the long forms of school-rooms, or on the round stool, to practise for hours on the piano-forte or harp, with strict injunctions to keep the body quite upright, and menaces of punishment, if they stoop or bend in the least; but the muscles must sometimes obey divine, instead of human laws, and when fatigued and weary in the erect posture, must gradually follow the Creator's law, and seek repose, by allowing the body to sink into an inclination to one side or the other, and, by laying the basis of lateral curvature, produce the reverse of what human wisdom intended. Thus, from too much anxiety about the elegant and proper development of the female figure, tyrant fashion, under the apparent sanction of custom, and the specious guise of education, has usurped the prerogatives of nature, has abolished the freedom of muscular action, and has presumptuously substituted the rules of art to direct the growth, and mould the form of the human figure, and direct its muscular powers!—Surely the Creator never intended these restraints; nature disdains them; and *he* who gave us powers to maintain the spine erect, also ordained its form to be bent, and its movements to be unfettered. Before man became subject to the influence of refined civilization, he enjoyed the privilege of free exercise of all his muscles, in the most untutored and unlimited manner, practised what attitudes he pleased, indulged in such sports and pastimes as he listed, and, when weary, assumed the recumbent posture he chose, without being shackled by the dictates of art; spinal deformity was then unknown to those who lived in a free state of nature, often called savage or barbarous.

The growth of the body, the proportions of form, the adjustment of the figure, the turn and directions of the bones, were left to the unerring hand of nature, and freedom of muscular movements. But, by the force of cruel fashion, nature is almost forsaken, and art is so preposterous and assuming as to offer dictates, and prescribe directions to form the body agreeably to its natural configuration; and parents, and proprietors of female seminaries or schools, are so silly and unreasonable as to submit to their guidance. Nay, this submission to the suggestions and dictates of art has even been made in one department of our government; and in a great national institution for the rearing of orphan children of soldiers, there is established the appointment of an exercise and figure master, with the pay of a captain in the army, who is to control and give a proper direction to the growth of the body, 'to teach the young idea how *to shoot*,' and elicit and confer tone on all their powers, by artificial exercises of particular muscles, in a numerous train of movements, and list of attitudes. Against these artificial habits and customs, wisdom has launched its mandates, genius has levelled its enlightening arguments, experience has pronounced its judgment, reason has fulminated its reproaches, wit has pointed its keen shafts of ridicule, and authority has proclaimed its interdictions; yet the folly of fashion increases, and its prevalence extends! In passing through the school-rooms of the seminaries for young ladies, what can be more appalling and preposterous, than to see the motionless figures of a long row of young ladies lying on inclined or horizontal planes of deal boards, 'like *patients* on a monument smiling at the white-washed ceilings,' or like the mute statues on tomb stones of those interred beneath, and from which we should hardly distinguish them, were it not for the occasional movements of the muscles of their (should be) brilliant eyes, or laughter-loving muscles of the face, whose situation defies control and eludes confinement. The conduct of boys' modern schools, is, also, exposed to serious objections, in common with the girls' schools. Boys and girls are too much shut up in small play-grounds, like prison yards, instead of being allowed to range the valley free, or girls have small gardens to walk in enclosed by high walls, like those of the asylums for the insane. What can be more contrary to nature's freedom of movement, than marching schools of boys and girls, in rank and file, like the boys at the military asylum, instead of allowing them to move at ease, in any place they prefer, or are prompted to?

Without digressing further, I would observe, instead of stiff stays, back boards, reclining boards, education chairs without backs, military marching, &c., let the girls and boys have no clothes or apparatus to limit their movements, and when weary, let them sit down on chairs with proper curved backs to support the spine, or lie down for rest, or, in fact, seek repose as they find most agreeable, when they are fatigued, or can no longer maintain an erect attitude conveniently. Let the girls have a large field or play-ground, let the boys, also, have the range of the country, within the sound of the school-bell. Let the girls engage in the games of battledoor and shuttlecock, skipping, dancing, and all that they can play at. Let the boys play at cricket, trap and ball, shinney, fives, skipping, running, coits, marbles, climbing trees, jumping, &c., and we shall not have many distortions of the spine; and, without intending to give offence, I will venture to express an opinion, that if the amount of a captain's pay was laid out for the use of the boys at the military asylum, in the purchase of cricket bats, trap and balls, skipping ropes, swings, shinney sticks, marbles, coits, &c., there would not be any occasion for exercise masters, or surgeons to cure deformities, unless arising from scrofula or accident."

The importance of watching the earliest manifestations of disease in the spinal marrow, is well shown in the following remarks on the "*Causes of Inflammation of the Spinal Cord.*"

"The remote causes of inflammation of the spinal cord, in the cases that are published, and in my practice, have been contusions on the spine; strains; tumours pressing on it; suppressed discharges, as of the ears, vagina and uterus; carious vertebræ; metastasis of inflammation; cold or suppressed perspiration. The immediate cause is inflammatory action of the blood-vessels of the medulla and membranes. When it is considered that the medulla spinalis is a continuation of both divisions of the brain, that the membranes are also continuous, and that the nerves of all the viscera and muscles below the head derive their origin from it, and that the due performance of their functions depends upon the healthy condition of their nerves, and that, in fact, all motion and sensation arise from it, it will not be a matter of surprise that the phenomena attending the disease, include derangements of all the principal organs and functions. The brain becomes affected, and the intellectual motions are deranged and imperfect; the muscular movements of the organs of sense and of the muscles supplied with nerves from the brain, are disordered and irregular. The heart is oppressed and affected with palpitation or increased action; the lungs and muscles subservient to respiration perform it with labour and difficulty. The abdominal and pelvic viscera become deranged in the manner that has been already described. All the muscles are affected with debility or paralysis, or irregular spasmodic actions like chorea, or with convulsive actions like epilepsy or tetanus. And these are the dreadful effects of inflammation of this small but important portion of our frame, which is so enclosed and defended as if it were intended to be impenetrable and inaccessible to disease."

We recommend Mr. Bampfield's work to our subscribers as one of the very best treatises on spinal diseases and their treatment extant.

